# Development and Evaluation of a “Speak-Up” Program for Patient Safety: A Virtual Reality-Based Intervention for Nursing Students

**DOI:** 10.3390/healthcare13222860

**Published:** 2025-11-11

**Authors:** Jeong Hee Jeong, Mi Jin Kim

**Affiliations:** 1 Department of Nursing Science, Kyungsung University, Busan 48434, Republic of Korea; loveu1105@ks.ac.kr; 2 Department of Nursing, Daegu Haany University, Gyeongsan-si 38610, Republic of Korea

**Keywords:** quality of care, nursing students, patient safety, quality and safety, nursing education

## Abstract

Background: This study aimed to develop and evaluate the impact of a virtual reality (VR)-based speak-up program for Korean nursing students to strengthen patient safety management competencies. A nonequivalent control group pretest–posttest quasi-experimental design was employed. Methods: Fifty-six fourth-year nursing students (28 each in the experimental and control groups) were enrolled. The experimental group participated in a VR-based speak-up program, while the control group engaged in a scenario-based speak-up program. Each program consisted of a single 150 min session. The experimental group completed the program individually using a head-mounted display device, whereas the control group participated in small-group sessions. Outcome measures included speak-up, sense of safety control, confidence in clinical decision-making, and patient safety management activities. Data were analyzed using SPSS 26.0, including the χ^2^ test, *t*-test, Mann–Whitney U test, Wilcoxon signed rank test and repeated measures analysis. Results: Speak-up performance significantly improved in both groups, but the differences between the groups were not significant. In contrast, sense of safety control, confidence in clinical decision-making, and patient safety management activities improved more in the control group, which engaged in discussion-based learning. These findings suggest that VR learning effectively strengthens individual assertiveness and behavioral readiness through immersive, self-directed experiences, whereas the discussion-based approach in the control group enhances collaborative reasoning and confidence related to patient safety. Conclusions: Integrating VR-based Speak-up education with existing learning methods can establish a stepwise program that enhances nursing students’ Speak-up competency and patient safety skills. This approach may bridge the gap between theory and practice, fostering nurses who actively promote patient safety.

## 1. Introduction

Speaking up is an important patient safety activity practiced by healthcare professionals when they voice their concerns to draw attention to recognized problems after realizing that a patient is unsafe, thereby preventing patient safety incidents (PSIs) [1]. Speaking up has a positive impact on decision-making, correction of human errors, development and improvement of programs for preventing PSIs [2], and overall enhancement of patient safety and care quality [3]. Nurses are well positioned to observe and manage early signs of problems related to patient safety [4]; therefore, when they notice suboptimal care, they should speak up to prevent PSIs.

However, among the primary causes of poor communication in hospitals, failure to speak up accounted for approximately 23% of incidents [5]. Nurses were found to be less likely to raise concerns, even though they are more likely than other healthcare professionals to recognize PSI-related problems [6]. In conflict situations, most nurses tend to adopt an avoidance attitude or passive submission, focusing on resolving the immediate issue rather than addressing the underlying causes [6]. Additional barriers to speaking up include fear of retaliation or negative reactions from others, concerns about appearing incompetent, perceptions that speaking up will not lead to change, and limited opportunities for effective information sharing [3,7].

In recent years, international reviews and patient safety organizations have continued to emphasize these persistent barriers, including hierarchical culture, fear of negative consequences, and insufficient communication training among nurses. The World Health Organization [8] and the OECD [9] have highlighted that developing structured education and psychologically safe learning environments is essential to empower healthcare professionals to speak up and prevent patient safety incidents. Most importantly, novice nurses who have not received training in speaking up demonstrate limited ability to recognize situations where patient safety is at risk and to report the problems they detect. In fact, they are more likely to remain silent rather than speak up, even when they become aware of adverse patient safety events [10].

In a hierarchical hospital structures, nursing students rarely have opportunities to speak up about patient safety concerns during their clinical practicum. Even when they recognize situations that may threaten patient safety, many remain silent due to their perceived low status and fears that speaking up could negatively affect their academic grades or relationships with supervisors [11]. Therefore, structured education is essential to help future nurses overcome this culture of silence and actively voice concerns to prevent PSIs [12]. Because learning to speak up requires more than a single exposure, continuous and repeated practice is needed to foster lasting behavioral change. Accordingly, incorporating speak-up training into undergraduate nursing curricula before nurses enter clinical settings may be more effective than introducing it after licensure [13].

Clinical simulation is widely used in patient safety education, as it allows replication of situations where safety may be compromised. However, in such team-based simulations, nursing students typically work under the supervision of an instructor, and opportunities to speak up are not equally distributed among all team members [14]. Scenario-based learning also enhances nursing performance through realistic case studies aligned with learners’ understanding, yet it remains limited in providing education on the evaluation phase, where nurses must assess changes in patient conditions and outcomes [15]. In contrast, virtual reality (VR) simulation offers a contactless learning environment that requires only access to a computer and provides immediate feedback without instructor involvement [13]. Through VR, students can independently practice speaking up across diverse clinical situations without social pressure or observation from others [16]. Moreover, VR enables full immersion in realistic environments that cannot be replicated through conventional simulation methods [17].

Beyond immersion, VR is particularly suitable for training the act of “speaking up” in hierarchical healthcare contexts because it minimizes the psychological barriers associated with authority gradients and fear of negative evaluation [3,6]. Unlike traditional team-based simulation, VR ensures that each learner independently voices concerns and makes decisions, thereby eliminating the tendency for more dominant participants to overshadow others [7,18]. Furthermore, VR enables repeated and individualized practice of voice behavior, which is essential for developing confidence and persistence in speaking up despite social or cultural hierarchies [13,19]. Such pedagogical advantages make VR a more appropriate modality than conventional simulation for cultivating this specific competency [17,20].

In addition, opportunities for repeated practice in speaking up are essential because pedagogical effectiveness varies depending on individual skill levels [13]. VR provides an effective methodology that allows nursing students to engage in repeated, self-paced simulation experiences and receive immediate feedback [19].

Speaking up represents the first step in patient safety management activities aimed at preventing PSIs [6]. To effectively speak up, learners must first develop a sense of safety control, recognizing situations that threaten patient safety and implementing appropriate responses [21]. Moreover, confidence in clinical decision-making is required to analyze patient information and select the most appropriate nursing actions [22].

Therefore, examining the effects of a VR-based speak-up education program on speaking-up behavior, sense of safety control, confidence in clinical decision-making, and patient safety management activities among nursing students can provide valuable insights. Speaking-up education for nursing students in Korea is unfortunately still at an early stage of development and has not yet been systematically incorporated into the nursing curriculum. While previous studies in Korea have mainly focused on the perceptions and experiences of registered nurses [23], little research has explored the development of speaking-up competence among nursing students. Internationally, simulation-based interventions have shown that practicum courses can improve students’ confidence in speaking up for patient safety [24] and enhance their communication skills [18]. However, few studies have investigated VR-based approaches for this purpose.

Although the concept of “speaking up” has been widely examined internationally, its systematic integration into undergraduate nursing curricula in Korea remains limited. To our knowledge, no study has addressed this educational gap by developing a structured program that enables nursing students to repeatedly practice speaking up in realistic clinical scenarios. The present study contributes to filling this gap by implementing a VR-based program that provides individualized, self-paced practice and immediate feedback on the adequacy of speaking-up behavior and subsequent nursing performance. This instructional design allows each participant to have a firsthand experience of voicing concerns in a psychologically safe, immersive environment—an approach that distinguishes this study from previous simulation-based interventions.

## 2. Methods

### 2.1. Research Design

This was a nonequivalent control group pretest-posttest quasi-experimental study involving nursing students, conducted to evaluate the impact of a VR speak-up program.

### 2.2. Objective

This study aimed to develop a VR-based speak-up program (hereinafter, VR speak-up program) for nursing students to strengthen patient safety management competencies and evaluate the impact of the program.

### 2.3. Hypotheses

The hypotheses developed according to the study objectives were as follows:

**Hypothesis 1.** 
*There is a difference in speak-up pre and post intervention between the experimental group exposed to the VR speak-up program (hereinafter, experimental group) and the control group that is not exposed to the VR program (hereinafter, control group).*

**Hypothesis 2.** 
*There is a difference in the sense of safety control pre and post intervention between the experimental group and control group.*

**Hypothesis 3.** 
*There is a difference in the confidence in clinical decision-making pre and post intervention between the experimental group and control group.*

**Hypothesis 4.** 
*There is a difference in patient safety management activities pre and post intervention between the experimental and control groups.*

### 2.4. Research Data

Fourth-year nursing students with at least one year of experience in clinical practicum from K University and D University in Korea were selected to participate in the study. As the VR speak-up program used scenarios based on clinical cases, participants needed to have a basic understanding of clinical settings and situations. Therefore, fourth-year students with at least one year of clinical experience were chosen as study participants.

Participants were quasi-randomly assigned to groups by university through a drawing lots method. Specifically, K University students were allocated to the experimental group and D University students to the control group. Although randomization was attempted at the institutional level, full individual randomization was not feasible due to practical constraints.

The curricula for undergraduate nursing students at both K University and D University conform with the standards accredited by the Korean Accreditation Board of Nursing Education. Thus, both universities have a clinical practicum course from the first semester of their third year of study in a variety of clinical settings, such as tertiary hospitals or general hospitals, indicating no significant difference in providing the practicum course between the two universities. Nursing students who volunteered to participate through the recruitment notice posted on the campus but expressed anxiety or repulsion to wearing head-mounted display (HMD) equipment and those who could not see the VR screen properly when their glasses were removed were excluded. After the screening, nursing students who consented upon hearing an additional explanation of the details of the study were included as the final participants.

For calculating the sample size required to conduct this study, G*Power 3.1.9 was used. In repeated measures analysis of variance (ANOVA), the conditions for calculation were as follows: significance level α = 0.05, power (1 − β) = 0.90, number of measurements = 2 times, correlation among repeated measures = 0.50, number of groups = 2, and medium effect size (*f*) = 0.25. Accordingly, the total sample size was 46, with 23 participants for each group, and by considering a dropout rate of 20%, the sample size was determined to be 28 participants for each group (a total of 56 participants). For sample size determination, as it was difficult to find a study investigating the impact of the speak-up intervention for undergraduate student nurses, so the effect size was calculated using G*Power 3.1.9 from the result of the study [25], which examined the speak-up effect for nurses. Since the speak-up effect size of the previous study [25] was *d* = 0.60, which was more than the medium effect size, the number of subjects was selected based on the medium effect size *f* = 0.25. We applied a more conservative estimate to ensure sufficient statistical power and robustness in the absence of directly comparable studies with undergraduate nursing students. As there were no dropouts during the intervention period, data from 28 participants each from the experimental and control groups were included in the final analysis.

### 2.5. Variable Selection

#### 2.5.1. Participants’ General Characteristics

The participants’ general characteristics comprised nine items in total, namely gender, age, VR experience, VR knowledge, necessity of VR-based educational content, expectations from VR-based education, confidence in VR-based education, anxiety about VR-based education, and usefulness of VR education in nursing performance.

#### 2.5.2. Speak-Up

Speak-up refers to healthcare professionals’ habit of raising concerns or objections for the benefit of both patient safety and care quality on recognizing risky or deficient actions, misdiagnosis, poor clinical judgment, rule breaking, and failure to follow standardized protocols by other members of healthcare teams [3]. Speak-up was developed by Premaux and Bedeian [26] and used by Sayre [25] in the study on nurses. It was used through the process of being translated into Korean and then back-translated into English. The Speak-up tool question was not interpreted differently in meaning or concept after translation into Korean, and it was found that there was not much difference from the previous sentence when translating Korean back into English by experts fluent in Korean and English. This measure comprises a total of five questions on whether the respondent speaks up for issues that may be controversial, and each question is assessed on a five-point scale; higher scores indicate a high level of speak-up performance. As for reliability, Cronbach’s α was 0.87 at the time of developing the measure and 0.75 in this study.

#### 2.5.3. Sense of Safety Control

Sense of safety control refers to an individual worker’s perception that influences work performance in relation to deriving safety outcomes [21]. The sense of safety control was developed by Anderson et al. [21], and used a tool translated into Korean by Kwon and Hwang [27]. The measure comprises seven questions on the perception of control for patient safety in a hospital’s organizational environment, with each question assessed on a five-point scale; higher scores indicate a higher sense of safety control. As for reliability, Cronbach’s α was 0.85 at the time of developing the measure; the value was 0.83 in Kwon and Hwang’s study [27] and 0.84 in this study.

#### 2.5.4. Confidence in Clinical Decision-Making

Confidence in clinical decision-making refers to a candidate’s confidence in performing a series of processes from data collection, interpretation of the meaning of data, analysis of relationships, selection of alternatives, inference, synthesis, and demonstration to provide patient care in clinical practice [22]. As for the measure of confidence in clinical decision-making, the Nursing Anxiety and Self-confidence with Clinical Decision-Making scale (NASC-CDM) developed by White [22] was translated and adapted by Yu et al. [28] to produce the KNASC-CDM, and this Korean scale was used in this study. The KNASC-CDM has four dimensions and a total of 23 questions. Dimension 1 is “listening fully and using resources to gather information” (8 questions), Dimension 2 is “using information to see the big picture” (7 questions), Dimension 3 is “knowing and acting” (5 questions), and Dimension 4 is “seeking information from clinical instructors” (3 questions). Each question is assessed on a six-point scale and higher scores indicate higher confidence in clinical decision-making. As for reliability, Cronbach’s α was 0.98 in White’s study [22], 0.93 in Yu et al.’s study [28], and 0.96 in this study.

#### 2.5.5. Patient Safety Management Activities

Patient safety management activities refer to measures adopted to prevent all errors of negligence, mistakes, and accidents that may occur during the delivery of healthcare services, regardless of whether the patient actually experienced any harm [29]. As for the scale of patient safety management activities, from the items listed in the Accreditation Standards for Hospitals, published by Korea Institute for Healthcare Accreditation [30], and the content described in the six International Patient Safety Goals (ISPGs), items that were closely relevant to this study were selected and included in a questionnaire to develop a scale for patient safety management activities. The scale had 12 questions in total, with 4 questions on accurate patient identification, 5 questions on communication among healthcare professionals, and 3 questions on accurate checking for surgery/procedure. Each question was assessed on a five-point scale, and higher scores indicated a higher level of patient safety management activities of nurses. In this study, Cronbach’s α was 0.85.

### 2.6. Data Analysis Method

The collected data were analyzed using the SPSS Win version 26.0. There were no missing values in the data collected.

Participants’ general characteristics were expressed and calculated as frequency, percentage, mean, and standard deviation. The inter-group homogeneity of general characteristics and dependent variables was analyzed using the X^2^ test and *t*-test. In the analysis, variables such as age, necessity of VR content, expectations from VR education, confidence in VR education, anxiety about VR education, usefulness of speak-up education, and Speak-up showed *p* < 0.05 according to the Shapiro–Wilk test, indicating that the variables were not normally distributed. Therefore, the Mann–Whitney U test was used to analyze the inter-group homogeneity for these variables.

The speak-up was not normally distributed, so it was analyzed using the Wilcoxon signed rank test (the change over time before and after the intervention), and the Mann–Whitney U test (the difference between the two groups) as a nonparametric test.

Repeated measures ANOVA was used for sense of safety control, confidence in clinical decision-making, and patient safety management activities before and after the intervention between the two groups. The assumption of equality of covariance metrics of other dependent variables was confirmed by Box’s test, and variance homogeneity was confirmed through Mauchly’s test of Sphericity. The variables did not meet the assumption of sphericity, so the Greenhouse-Geisser value was reported among the results of with subjects effects.

Speak-up adequacy and nursing performance evaluation of the experimental group was conducted by one researcher, and the evaluation criteria are described in ‘Section 3.2.2 Development of algorithm and interface configuration’. Results were calculated as frequencies and percentages.

## 3. Program Development

### 3.1. Program Purpose

This program aims to guide undergraduate student nurses to recognize situations where PSIs may occur and to practice speak-up to prevent PSIs.

### 3.2. Program Development Process

In this study, the author developed the VR speak-up program for the experimental group between January 2022 and February 2023. During this process, the author’s team received help from an expert for VR production and advice. Details of the development process are as follows.

#### 3.2.1. Development of Scenarios for Nursing Pracitce Requiring Speaking Up and Assessment of Validity of the Developed Scenarios

In this study, content development was implemented based on the author’s previous work [31]. A total of 10 scenarios were developed from the patterns and cases of frequent PSIs that were derived from the interviews on the speak-up experiences of 12 nurses having no less than 10 years of clinical experience in patient safety management from three university hospitals and two general hospitals in B city (Table 1). The categories for the scenarios were medication, blood transfusion, and surgery/procedure, and the VR play time per scenario was approximately 5 min. Each scenario comprised a flow in which patient safety was maintained or PSI occurred depending on whether the participant recognized the problem situation and spoke up as an adequate response to the situation. Validity assessment was performed for the developed scenarios by two senior nurses responsible for training nurses at a tertiary hospital. The following aspects of the scenarios were modified or supplemented in terms of content or structuring of the situation: modification of the content to be communicated upon reporting, change in the medication dose for a pediatric patient, change in order for tests, addition of patient identification procedure before administration of medication, and addition of procedure for checking surgical site markings.

#### 3.2.2. Development of Algorithm and Interface Configuration

**Figure 1:**
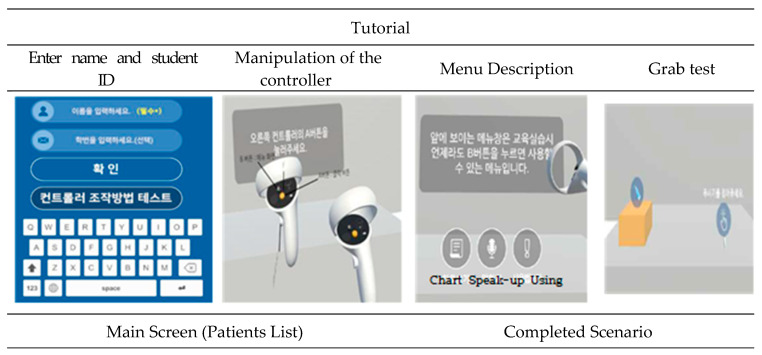

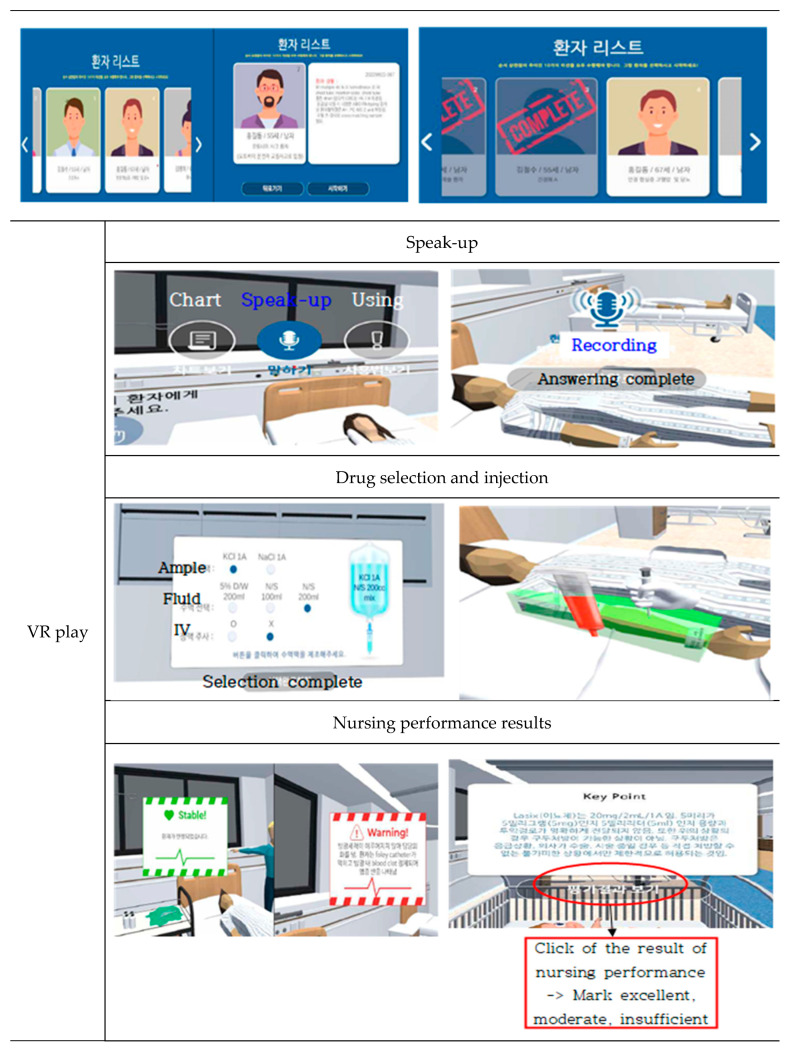
VR interface.

Prior to starting the VR speak-up program, a tutorial was provided after including a preparation process to aid in launching the program, which included the following: introduction to the program’s learning goals, guidance on how to operate the controller and its functions, picking up objects, a test on medication and speak-up willingness, and entering the student’s name and registration number. Thus, the process helped participants to familiarize themselves with the program. Subsequently, depending on the difficulty level, the scenarios were classified into high, medium, or low, and it was arranged that a student can start from the “low” level of difficulty. After a student completed a scenario, it would automatically disappear with the task marked as “complete.” (Figure 1).

The process of evaluation of speak-up adequacy performed during VR play was as follows. The result of speak-up adequacy was categorized into one of the following three levels: adequate, inadequate, or did not speak-up. The researcher rated the speak-up adequacy in a separate administrator mode soon after the participant spoke up so that the VR flow could be changed. Among the three levels, “inadequate” was marked when the participant’s opinion was not appropriate for the situation or when the opinion was not expressed clearly. In addition to evaluating speak-up adequacy, when one scenario was completed, the result of nursing performance related to maintaining patient safety or PSI occurrence was assessed and displayed on the participant’s VR screen in one of the following three levels: excellent, moderate, or insufficient. If there were no problems in providing nursing care and patient safety was maintained, then the nursing performance was marked as “excellent”; however, when a problem was observed in the process, it was rated as “moderate,” and when a PSI occurred, nursing performance was rated as “insufficient.” Before playing the next scenario, the participant was given some time to check his/her own nursing performance result so that each participant could think about any problems about their speak-up adequacy and problems during the process of nursing care provision. After reflection, the participant pressed the Scenario Play Start button; thus, improvement in speak-up could be achieved through the process of repetition and feedback (Figure 1).

#### 3.2.3. System Development and Evaluation of Validity

Based on the content and process of the VR program described above, the VR speak-up program was developed with the help of a VR production expert, and when the author suggested areas for improvement, modification, or supplementation after testing, the expert reflected on the suggestions in the development process. The HMD used for the program was Oculus Quest2 (Meta), which comprises a VR headset and Quest touch controller. If graphic displays are created according to the scenario flow chart and participants view the graphics only during their participation, it may result in reduced concentration and learning effect. Therefore, all nursing activities, such as patient identification, preparation for medication and blood transfusion, administration of medication, blood transfusion, and speak-up were performed individually by manipulating the controller. For the developed VR content, heuristic evaluation was performed by four professors in nursing using the heuristic evaluation scale for the VR systems developed by Murtza et al. [32], and usability assessment was performed by three third-year nursing students using the System Usability Survey developed by Brooke [33]. Based on the feedback during the evaluation and assessment, the following problems were identified and modified or improved accordingly: issue of disconnection in mirroring that shows the participant’s VR play status through the administrator mode, lack of explanation on the functions of controller buttons and preliminary testing of how to use the controller, system crash during VR play, and error of not allowing input in the administrator mode.

#### 3.2.4. Structure of the VR Speak-Up Program

The VR speak-up program comprised one session and three stages for a total of 150 min (Table 2). From the perspective of the VR speak-up program usability assessment, continuous playing of the scenarios in a single run increased a participant’s immersion in situations, the participant better achieved the intended purpose of the program, and a total of one session was determined to be appropriate for the VR program. The VR program cannot be played by multiple participants at the same time with one HMD device, and individual interventions were planned because the author or the research assistant had to intervene and provide help for each participant. As for the schedule of individual interventions, a maximum of two participants could undergo the intervention at the same time, and a maximum of four participants could receive the intervention per day. Before VR play, an overview of the VR play program was first provided to help the participants adapt to the program, and time was allocated for debriefing and sharing their learning experience after the VR play.

### 3.3. Program Details

#### 3.3.1. Experimental Group: VR Speak-Up Program

The VR speak-up program applied to the experimental group comprised three steps. Step 1 was 30 min long, and explanations were provided in a lecture style on the purpose of the program, the concept of VR, how to use the VR headset and controller, an explanation of the content introduced in the tutorial, the direction of scenario progression, and the evaluation method. Explanations were given so that students could comprehend the VR content and possess accurate information about the role they should play in the program. Handouts on the related information and references and the real VR equipment were used during Step 1. In Step 2, the VR play proceeded for 50–60 min per participant. As each scenario had a running time of approximately 5 min, 10 scenarios (Table 1) were played out and arranged in the order of level of difficulty as low -> medium -> high. Owing to differences in the proficiency of handling equipment by individual participants, 50–60 min of time was allocated for the VR play. During Step 2, the participant wore a VR headset, used the controller, and played the role of a nurse in VR, solving problems by speaking up in situations that posed risks to patient safety. After a 10 min break, the last step, Step 3, lasted 50 min, and participants discussed their experiences with the program. Thoughts on patient safety, the importance of speaking up, barriers to speaking up, strategies for promoting speaking up, using the program for learning, and satisfaction with the program were shared during the discussion, and the program session was concluded.

#### 3.3.2. Control Group: Scenario-Based Speak-Up Program

In the control group, seven subgroups with four participants each were formed to implement the scenario-based speak-up program. As with the experimental group, the program was divided into three steps. The scenario used for the control group was the same as the one applied to the experimental group, and the difference between the two groups was the presence of the VR play. Step 1 lasted 30 min, and the purpose and process of the program, evaluation method, and definition of speak-up were explained using handouts. A total of three scenarios, one each for high, medium, and low levels of difficulty, were randomly provided to each subgroup. Step 2 lasted 60 min, and in the order of the level of difficulty low -> medium -> high, the scenarios were analyzed and discussed for each subgroup. A question-and-answer session was held in which the author asked about a situation in the scenario and the participants responded on how they would speak up in each situation. Step 3 lasted 50 min, and the participants shared their thoughts on the same content as the experimental group, and the program session was concluded.

### 3.4. Procedure

This study was conducted with participants who were fourth-year nursing students at K University and D University from 12 April to 10 July 2023, and details of the study process are as follows.

#### 3.4.1. Preparation for Program Operators and Research Assistants

The author and research assistants were the program operators. Two nursing graduates were appointed as research assistants, and the researcher fully explained the overview of the VR Speak-up program, how to use it, the contents of the survey questionnaire to the research assistants, and trained data collection technology to minimize errors between measurers. In addition, prior to data collection, the author educated the research assistants on ensuring ethical considerations and maintaining data confidentiality in the study.

#### 3.4.2. Program Implementation and Survey

The developed VR speak-up program was implemented for the experimental group, and the scenario-based speak-up program was implemented for the control group, both for a total of one session (150 min) from 8 May to 10 July 2023. The program for the experimental group proceeded with a maximum of four participants in one day, and the control group’s schedule was arranged into small subgroups. In the case of the control group, the VR speak-up program was provided to those who expressed their wish to receive the program after a post-intervention survey.

The pre-intervention survey was conducted between 12 April and 26 April 2023, after the participants filled out the consent form for study participation, and the two research assistants collected data using a structured questionnaire. The questionnaire took approximately 20 min to complete, and the author provided guidance to facilitate the data collection process. The post-intervention survey was conducted immediately after, that is, at the end of the intervention, using the same method as the pre-intervention survey.

## 4. Results

### 4.1. Participant Characteristics and Test of Homogeneity

On comparing the general characteristics of the participants, there were no statistically significant differences between the two groups. Furthermore, on comparing the study’s dependent variables, namely, speak-up, sense of safety control, confidence in clinical decision-making, and patient safety management activities, there were no statistically significant differences between the two groups. The pre-test results of inter-group homogeneity for the general characteristics and dependent variables between the experimental group and control group verified the homogeneity of the two groups (Table 3).

### 4.2. Hypotheses Testing

Hypothesis 1: “There is a difference in speak-up pre and post intervention between the experimental group and control group.” Nonparametric tests were performed, and the change over time before and after the intervention (Z = −4.99, *p* = <0.001) were significant, but the difference between the two groups (Z = −1.61, *p* = 0.108) was not significant. Hypothesis 1 was partially supported.

Hypothesis 2: “There is a difference in the sense of safety control pre and post interventions between the experimental group and control group.” Statistically significant differences were found in the difference between the two groups (F = 27.11, *p* = <0.001), the change over time before and after the intervention (F = 21.65, *p* = <0.001), and the interaction between the group and time (F = 7.04, *p* = 0.010); thus, Hypothesis 2 was supported.

Hypothesis 3: “There is a difference in confidence in clinical decision-making pre and post intervention between the experimental group and control group.” Statistically significant differences were found in the difference between the two groups (F = 12.13, *p* = 0.001), the change over time before and after the intervention (F = 41.58, *p* = <0.001), and the interaction between the group and time (F = 4.18, *p* = 0.046); thus, Hypothesis 3 was supported.

Hypothesis 4: “There is a difference in the patient safety management activities pre and post intervention between the experimental group and control group.” Statistically significant differences were found in the difference between the two groups (F = 6.66, *p* = 0.013), the change over time before and after the intervention (F = 111.09, *p* = <0.001), and the interaction between the group and time (F = 13.24, *p* = <0.001); thus, Hypothesis 4 was supported (Table 4).

### 4.3. Speak-Up Adequacy and Nursing Perfomance of the Experimental Group Based on the Level of Scenario Difficulty

After dividing the scenarios into high, medium, and low according to the level of difficulty, the VR speak-up program was implemented starting from the “low” level scenarios, and the evaluation results of speak-up adequacy and nursing performance of the experimental group are presented in Table 5.

The proportion of “adequate speak-up” was 10.7–46.4% for the “low” level scenarios, 10.7–57.1% for the “medium” level, and 17.9–53.5% for the “high” level scenarios. The proportion of “did not speak-up” was 39.3–82.1% for the “low” level scenarios, 7.2–85.7% for the “medium” level scenarios, and 25.0–39.3% for the “high” level scenarios. As a result of the nursing performance evaluation, the ratio of “excellent” performance was 17.9–60.7% for the “low” level scenarios, 7.2–75.0% for the “medium” level scenarios, and 17.9–50.0% for the “high” level scenarios.

## 5. Discussion

In nursing education, although the importance of effective communication among healthcare professionals to prevent PSIs has been repeatedly emphasized, structured programs that allow nursing students to practice speaking up remain limited [12]. Against this background, this study developed and evaluated a VR-based Speak-up program tailored for undergraduate nursing students.

The analysis revealed no statistically significant difference in speak-up performance between the experimental and control groups (*p* = 0.108). Nevertheless, both groups showed improvement following the intervention, suggesting that technology-enhanced learning can still contribute to strengthening nursing students’ competencies related to patient safety. This result is similar to previous findings showing that simulation-based learning in immersive and interactive environments strengthens students’ assertive communication and proactive voice behaviors [18,34]. Likewise, a mobile application-based VR education program has been shown to improve nursing students’ knowledge, attitudes, and confidence in patient safety competencies [20].

In this study, the experimental group was asked to identify unsafe conditions, such as incorrect surgical site markings, and explicitly state, “The surgical site is on the left, not on the right.” Initially, students found this task difficult because speaking up in a virtual setting without peer or instructor support required independent judgment and professional confidence [7]. Early responses were often characterized by hesitation or silence, but through repeated exposure to increasingly complex VR scenarios, participants improved their ability to recognize risks and articulate concerns [16]. The immersive and realistic features of the VR environment provided an authentic yet psychologically safe space in which students could confront challenges, reflect on their communication strategies, and adapt their responses in real time [35]. Through this iterative process, they internalized assertive communication skills and gradually overcame the emotional tension and hierarchical barriers that often discourage students from voicing concerns in clinical settings [24].

The control group also demonstrated meaningful progress through discussion-based learning. Participating in structured discussions enabled students to collaboratively analyze clinical situations, share perspectives, and practice expressing opinions in a supportive environment. This suggests that both immersive VR learning and reflective discussion foster communication competence in different yet complementary ways. Integrating VR-based Speak-up training alongside discussion-oriented instruction may therefore provide a balanced and effective educational approach for cultivating confident and proactive nurses who advocate for patient safety.

Beyond Speak-up performance, both groups improved in sense of safety control, confidence in clinical decision-making, and patient safety management activities; however, the control group showed greater gains in these areas. Case-based learning applied to the control group has been shown to be an effective educational method for improving academic achievement and case-analysis skills among medical students [36]. Its effectiveness has also been demonstrated in patient safety education for nursing students [37]. Through small-group discussions, students engaged in shared analysis and collaborative reasoning, which enhanced their confidence and awareness of patient risks. Many reported, “I developed confidence when group members came together to solve problems,” and “Working through the case with peers helped me organize my thoughts and recognize patient risks I might have overlooked.” Such experiences reflect the benefits of interactive discussion, which promotes self-efficacy, shared responsibility, and a deeper understanding of patient safety principles [37].

In contrast, the experimental group participated in the VR-based speak-up program, which required each student to act independently by identifying risks, making decisions, and speaking up without group input. This design ensured full engagement and minimized the passivity sometimes observed in group discussions. Throughout the ten VR sessions, students identified cues indicating potential threats to patient safety, analyzed the clinical situations [38], and recognized that speaking up and performing appropriate nursing interventions could prevent patient safety incidents. The built-in feedback system further enabled participants to immediately evaluate their responses and reflect on their clinical decision-making processes. Repeated practice across progressively challenging scenarios strengthened their persistence and confidence in speaking-up behaviors [13,19]. Therefore, while discussion-based learning is particularly effective for enhancing collaborative reasoning and safety management, VR learning uniquely fosters individual assertiveness and preparedness to respond to patient safety risks.

Among participants in the experimental group, reflections revealed contrasting experiences. Some students stated, “I felt as if I became a real novice nurse,” and “I would pay more attention to patient safety when I begin clinical practice,” indicating that the VR experience enhanced their awareness and professional engagement. In contrast, others admitted, “If this had been a real situation, I might have hesitated and failed to speak up,” pointing to barriers such as hierarchical pressure and limited clinical knowledge that undermine confidence in judgment [24]. These divergent responses suggest that while VR training can strengthen recognition of safety risks and motivation to act, hesitation may persist when students lack sufficient experience or conceptual understanding to support their assertions. Therefore, repeated, structured, and individualized training during prelicensure education is essential to help students overcome hierarchical barriers [19]. Such training also enables them to build the knowledge base and confidence necessary to voice concerns when patient safety is at risk.

Taken together, these findings suggest that VR Speak-up training and discussion-based learning play distinct yet complementary roles in fostering patient safety competence. VR promotes individual accountability and assertive communication through immersive, self-directed experiences, whereas discussion-based learning strengthens teamwork, reflection, and shared problem-solving. Therefore, a hybrid educational model that combines VR-based experiential learning with discussion-oriented and clinical instruction may offer the most comprehensive and effective strategy for preparing nurses who can communicate assertively and collaborate effectively to ensure patient safety.

Several limitations should be noted. First, because universities rather than individuals were assigned to intervention and control groups, institutional bias cannot be excluded. Furthermore, the experimental group worked individually while the control group engaged in team-based learning, which may have interacted with the group assignment to produce additional confounding effects. Future studies should employ random assignment and control for learning modality to minimize institutional and instructional bias. Second, the sample size was calculated based on effect sizes reported in previous studies; while a conservative estimate was applied, the justification remains limited by the absence of directly comparable research on undergraduate nursing students. Replication with larger and more diverse samples is needed to provide more robust evidence. Third, while both groups improved significantly, the interpretation of VR’s added benefits should be understood in light of the substantial gains observed in the control group. Fourth, “speak-up adequacy” was evaluated by a single researcher in administrator mode, and the rating influenced the subsequent flow of the program. This may have introduced rater bias and expectancy effects. Future research should adopt blinded assessment procedures or employ multiple independent raters to establish inter-rater reliability and minimize potential bias. Finally, the experimental group worked individually in the VR program, whereas the control group engaged in team-based activities. This introduces a potential confounding factor, as improvements may reflect not only the intervention format (VR vs. scenario-based) but also differences between solo and team learning environments.

Therefore, the findings should be interpreted with caution, and future research should control for learning modality to better isolate the specific contribution of VR. Importantly, this also emphasizes that scenario-based education remains highly valuable, while VR’s unique contribution lies in ensuring individualized practice and reducing the influence of group dynamics. Future studies should investigate the complementary use of VR and group-based training to maximize learning outcomes.

## 6. Conclusions

This study developed and evaluated a VR-based Speak-up program designed to enhance nursing students’ ability to recognize and respond to patient safety risks. Both the experimental and control groups demonstrated improvement in Speak-up performance, suggesting that immersive VR training and discussion-based learning each contribute to strengthening assertive communication and proactive safety behaviors in complementary ways.

Discussion-based learning, which fosters collaborative reasoning and shared reflection, appears particularly effective in developing confidence and safety management skills. In contrast, VR learning uniquely promotes independent judgment, self-efficacy, and readiness to act through individualized, psychologically safe, and repetitive practice. Together, these approaches contribute to the development of comprehensive patient safety competence.

A tiered educational model that begins with discussion-based sessions to build conceptual understanding, followed by VR practice to reinforce behavioral application, may optimize learning outcomes. Incorporating structured feedback and debriefing after each VR session can further strengthen learners’ confidence and facilitate the transfer of skills to clinical settings.

## Figures and Tables

**Table 1:** Scenario Overview.

No.	Category	Level of Difficulty	Contents
1	Medication	low	A 68-yo female patient admitted; continuous bladder irrigation was ordered with 3L normal saline via a Foley catheter because of hematuria. During the handoff, the off-going nurse said “use 3L NS for irrigation at 10 gtt,” but the on-coming nurse misunderstood and started preparing for IV.
2	Medication	medium	An infant diagnosed with UTI (4 months, 4.4 kg) ordered Netilmicin (150 mg/1.5 mL) 1Ⓐ IV. The primary nurse checked the order and started preparing for medication administration.
3	Medication	medium	A 55-yo male patient diagnosed with liver cirrhosis, ordered KCL 1Ⓐ + 5% D/W 200 cc, but the medicines are incorrectly mixed. The primary nurse needs to attend to an urgent call from another patient and is asking a coworker to administer the medication.
4	Medication	high	A 44-yo male patient, with a history of laparoscopic appendectomy POD #2. The patient is complaining of abdominal pain and is asking for pain medicines. The primary nurse notified the attending provider. The attending provider is in the middle of a procedure and gave verbal orders with only the name of the pain medication. The primary nurse is preparing for medication administration.
5	Medication	high	A 67-yo male patient, admitted with diagnosis of stable angina and history of HTN and DM. Current orders are I/O check q 8 hrs. At 6 am, the previous day’s total I/O was reported as 2040/810 mL, with the patient showing a 2 kg weight gain. The nurse notified the attending provider. After rounds, the attending provider gave a verbal order: “Give 5 of Lasix.” The primary nurse is preparing for medication administration.
	Flow chart example	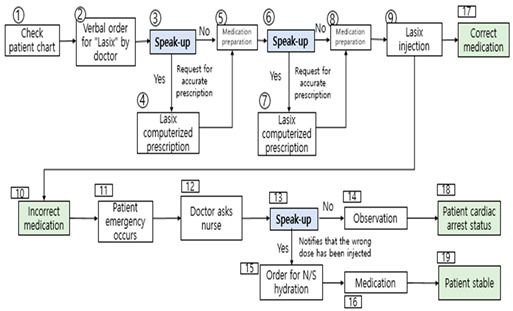	
6	Medication	high	Gil-Dong Ko (M/55). Admitted to Bed 5 in room 6803 after a motorcycle accident. Patient is receiving tazoperan 4.5 G I vial q 6 hr IV. A few days later, the patient was moved to a different bed, and another patient named Gil-Dong Hong is transferred to Bed 5 from the ICU. Gil-Dong Hong is allergic to tazoperan. The nurse, unaware of the change in beds for patients, confirmed the name “Gil-Dong” and prepared to administer tazoperan.
7	Blood transfusion	medium	A 55-yo man is admitted after a motorcycle accident. The patient had Rt multiple rib fx/c hemothorax, and an Rt chest tube was inserted. The patient is showing heavy chest tube drainage, and CBC showed Hb of 7.8. ABO Rh typing performed at the ED was Rh+ A. Provider’s orders were: “PC 400 2-units, cross-matching sampling before transfusion.” The nurse chose the sample bottle and went to the patient.
8	Blood transfusion	medium	A 60-yo male patient, blood type AB+ with no medical history. The patient fell from a 5 m height and sustained multiple fractures. The patient underwent emergency surgery and was admitted to the ICU. Hb was 6.9 g/dL. Provider put in orders for “packed RBC 320 2-unit transfusion.” The nurse must begin the second bag but is preparing for it alone because the ICU is busy, and all the nurses are occupied.
9	Surgery/ Procedure	low	A 40-yo female patient, being prepared for PS surgery due to a blow-out fracture. The surgical site is marked as Lt. on both the consent form and surgery schedule. Even during time out, everyone thought the surgery site was OS. However, on the outpatient record, the surgery is specified as OD surgery.
10	Surgery/ Procedure	low	A 55-yo male patient underwent a permanent pacemaker placement 5 years ago due to SSS (sick sinus syndrome). He recently developed severe back pain and was admitted to the NS. An order for MR L-spine was placed, and the patient was taken for MRI.

**Table 2:** Virtual Reality-Based Speak-up Program.

Step	Contents	Time (min)
Step 1. Introduction	- Introduction to program purpose and equipment usage method - Description of scenario progression direction - Present evaluation method	30
Step 2. VR play	- Check wearing equipment and linking with administrator mode - VR playing	50–60/per person
Break Time		10
Step 3. Debriefing	- Summary of opinions on speak-up - Share learning experience	50

**Table 3:** Homogeneity Test of General Characteristics and Study Variables in Pre-Test between Experimental and Control Groups (*N* = 56).

Variables	Range	Exp. (*n* = 28)	Con. (*n* = 28)	χ^2^/z/t	*p*
		*n* (%) or *M (SD)*	*n* (%) or *M* (*SD*)		
Gender				0.16	>0.999
Men		3 (10.7)	4 (14.3)		
Women		25 (89.3)	24 (85.7)		
Age (yrs)	21–25	21.96 (0.99)	22.14 (.08)	−0.65	0.516
VR experience(time)					
0		14 (50.0)	13 (46.4)	0.09	0.956
1		9 (32.1)	10 (35.7)		
≧2		5 (17.9)	5 (17.9)		
VR knowledge					
None		7 (25.0)	9 (32.1)	0.35	0.554
Commonly		21 (75.0)	19 (67.9)		
Necessity of VR content	2–10	7.64 (1.39)	7.54 (1.90)	−0.01	0.993
Expectations from VR education	5–10	8.39 (1.26)	7.93 (1.72)	−0.81	0.421
Confidence in VR education	2–9	5.00 (1.72)	5.64 (1.64)	−1.48	0.140
Anxiety about VR education	1–9	4.79 (2.04)	4.43 (2.44)	−0.56	0.578
Usefulness of VR education	4–10	8.07 (1.41)	7.96 (1.73)	−0.34	0.893
Speak up	8–22	16.43 (2.17)	16.82 (3.10)	−0.90	0.370
Sence of safety control	18–34	25.89 (2.42)	27.57 (3.73)	−2.00	0.052
Confidence in clinical decision-making	50–117	88.32 (16.18)	93.68 (16.12)	−1.24	0.220
Patient safety management activities	34–54	48.50 (4.57)	47.86 (5.27)	0.49	0.628

Note. VR = virtual reality; Exp. = experimental group; Con. = control group; M = Mean; SD = Standard deviation; yrs = years.

**Table 4:** Outcome of Speak-up Training Program (*N* = 56).

**Variables**	**Time**	**Exp. (*n* = 28)**	**Con. (*n* = 28)**	**Sources**	***F*** ***/Z***	***p***	**η** **p^2^**
		***M* (*SD*)**	***M* (*SD*)**				
Speak-up	Pre	16.43 (2.17)	16.82 (3.10)	Group	−1.61	0.108	
	Post	19.46 (2.17)	18.25 (2.58)	Time	−4.99	<0.001	
Sence of safety control	Pre	25.89 (2.42)	27.57 (3.73)	Group	27.11	<0.001	0.334
	Post	26.93 (2.93)	31.36 (2.44)	Time	21.65	<0.001	0.286
				G × T	7.04	0.010	0.115
Confidence in clinical decision-making	Pre	88.32 (16.18)	93.68 (16.12)	Group	12.13	0.001	0.183
	Post	99.25 (13.97)	114.75	Time	41.58	<0.001	0.435
			(11.40)	G × T	4.18	0.046	0.072
Patient safety management activities	Pre	48.50 (4.57)	47.86 (5.27)	Group	6.66	0.013	0.110
	Post	53.75 (3.68)	58.64 (2.87)	Time	111.09	<0.001	0.673
				G × T	13.24	<0.001	0.197

Note. Exp. = experimental group; Cont. = control group; M = Mean; SD = Standard deviation; ηp^2^ = partial eta squared; G × T = group × time.

**Table 5:** Speak-Up Adequacy and Nursing Performance Results for Each Scenario in the Experimental Group (*N* = 28).

Scenario Difficulty	Scenario No.	Speak-Up *n* (%)			Nursing Performance *n* (%)		
		Adequate	Inadequate	Did Not Speak-Up	Excellent	Moderate	Insufficient
Low	1	3 (10.7)	2 (7.2)	23 (82.1)	5 (17.9)	0 (0.0)	23 (82.1)
	9	13 (46.4)	4 (14.3)	11 (39.3)	17 (60.7)	0 (0.0)	11 (39.3)
	10	4 (14.3)	3 (10.7)	21 (75.0)	7 (25.0)	0 (0.0)	21 (75.0)
Medium	2	3 (10.7)	1 (3.6)	24 (85.7)	2 (7.2)	3 (10.7)	23 (82.1)
	3	16 (57.1)	5 (17.9)	7 (25.0)	21 (75.0)	0 (0.0)	7 (25.0)
	7	16 (57.1)	10 (35.7)	2 (7.2)	16 (57.1)	11 (39.3)	1 (3.6)
	8	6 (21.4)	0 (0.0)	22 (78.6)	6 (21.4)	22 (78.6)	0 (0.0)
High	4	5 (17.9)	16 (57.1)	7 (25.0)	5 (17.9)	16 (57.1)	7 (25.0)
	5	10 (35.7)	11 (39.3)	7 (25.0)	9 (32.2)	3 (10.7)	16 (57.1)
	6	15 (53.5)	2 (7.2)	11 (39.3)	14 (50.0)	1 (3.6)	13 (46.4)

## Data Availability

The data are not publicly available due to privacy/ethical restrictions.
